# Ochronosis of hip joint; a case report

**DOI:** 10.1186/1757-1626-2-9337

**Published:** 2009-12-16

**Authors:** Babak Siavashi, Mohammad J Zehtab, Ehsan Pendar

**Affiliations:** 1 Department of Orthopedic surgery, Sina hospital, (Imam Khomeini street), Tehran 11367-46911, Iran

## Abstract

**Background:**

Ochronosis is connective tissue manifestation of Alkaptonuria. Joint involvement specially hip and knee destruction is seen. The cartilage is pigmented and destroyed. It is interested for both pathologists and orthopedic surgeons.

**Case Presentation:**

A 54 years old woman with hip fracture after simple falling is candidate for surgery, but, after skin and subcutaneous incision over deep fascia there was dark blue pigmentation which continues toward hip joint. After biopsy of soft tissues and bone, in another operation, we replace hip joint.

**Conclusion:**

In this case, besides of cartilage destruction of hip joint, there was a lythic lesion of neck of femur which causes pathologic fracture of hip joint. We planned Total hip replacement instead of bipolar for her because of cartilage damage of acetabulum.

## Background

Ochronosis is the connective tissue manifestation of alkaptonuria, a defect in the metabolism of homogentisic acid (HGA) caused by autosomal recessive mutations of the HGO gene on chromosome 3q [1].

Homogentisic acid oxidase is responsible for the turnover of homogentisic acid (HGA) during the course of phenylalanine and tyrosine catabolism [2]. HGA accumulates and is polymerized into a blue-black pigment that ultimately is deposited in the skin, cartilage, and collagenous tissues. Specifically, pigment deposition can be seen in skin, bones, articular cartilages, synovial membranes, lungs, heart endocardium and valves, and kidneys [3]. The accumulation eventually causes severe degeneration of the spine and peripheral joints which may clinically simulate common arthritic disorders [4]. Alkaptonuria is a rare disease affecting one in 250,000-1 million people [5,6].

The most common clinical feature of ochronosis is ochronotic arthritis[6]. Other common presentations of ochronosis are:

Darkening of urine with exposure to air or reducing agents[7].

Thoracolumbar spine disc herniation, porotic vertebral bodies; osteophyte formation [8,9].

Hip and knee pain are due to cartilage degeneration, and in the future joint space narrowing, and sclerosis develop[2,10,11].

Cardiac valve calcification and stenosis, coronary artery calcifications [3,12-14].

Renal and prostatic stones [1].

Ochronotic pigmentation of sclera and ear cartilage[4,14].

Ruptures of tendons(patellar and Achilles tendons)[6,15].

Ochronosis has a classic triad: 1) degenerative arthritis; 2) ochronotic pigmentation; and 3) urine that turns black upon alkalinization. Confirmatory tests for diagnosis are chromatographic, enzymatic, or spectrophotometric determinations of HGA.

Currently, symptomatic treatment of the complications of alkaptonuria is the only option [1]. A symptomatic approach including treatment of pain, physiotherapy, chiropractic care, and education of the patient for a home exercise program is the treatment of choice[4].

Alleviation of pain and significant increases in activity has been achieved with total joint replacement of the hips, knees, elbows and shoulders[2]. A successful treatment for tendon ruptures due to ochronosis is primary repair[6]. High-dose vitamin C decreases urinary benzoquinone acetic acid, but has no effect on HGA excretion and, moreover, no credible studies have shown that treatment with vitamin C is clinically effective. Nitisinone, a potent inhibitor of 4-hydroxyphenylpyruvate dioxygenase, dramatically reduces production and urinary excretion of homogentisic acid[14]; however, the effectiveness of Nitisinone in treating ochronosis remains unknown.

## Case Presentation

**Figure 1:**
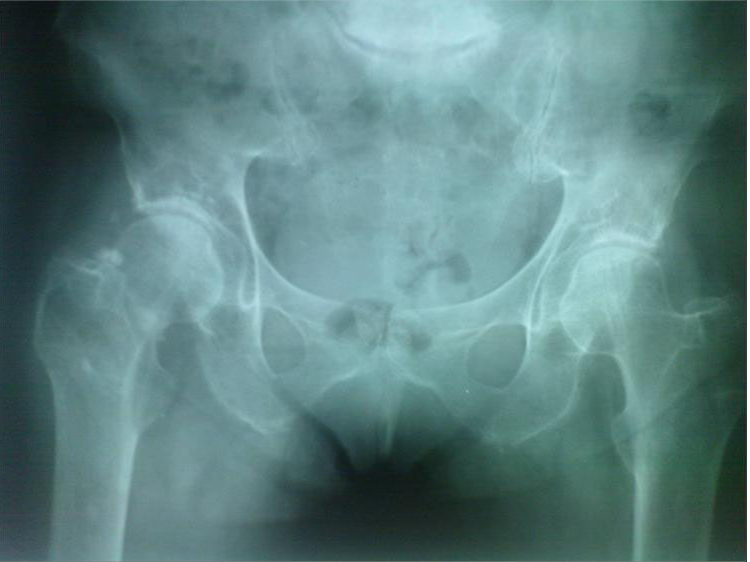
**Anteroposterior x-ray view of pelvis shows right femoral neck fracture**. X Ray, after falling of patient.

Our case here is related to a 54-year-old Iranian woman who was referred to emergency room of Sina hospital. She was feeling pain in her right hip after a simple falling, and could not bear weight on her leg. The anteroposterior and lateral radiography of hip joint showed a fracture of neck of the femur (fig. 1).

**Figure 2:**
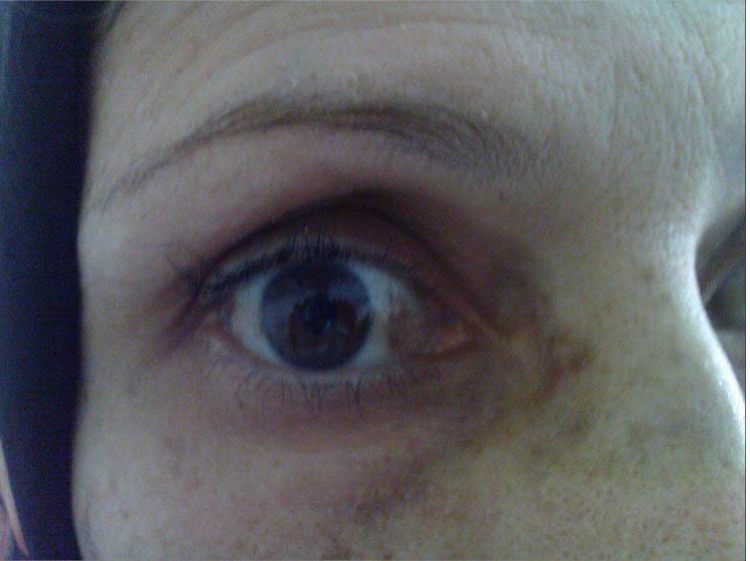
**Ochronotic pigmentation of the sclera**. Photography of patient's eye, after admission.

Because of a lucency at the base of femur neck, we came to the conclusion that a pathologic fracture had occurred at that region. There was no abnormality in laboratory data of the patient, and she had no other underlying disease except a chronic low back and hip pain. Roentgenogram of the spine showed space narrowing and calcification of the intervertebral discs. The patient had also ochronotic pigmentation of the sclera (fig. 2).

**Figure 3:**
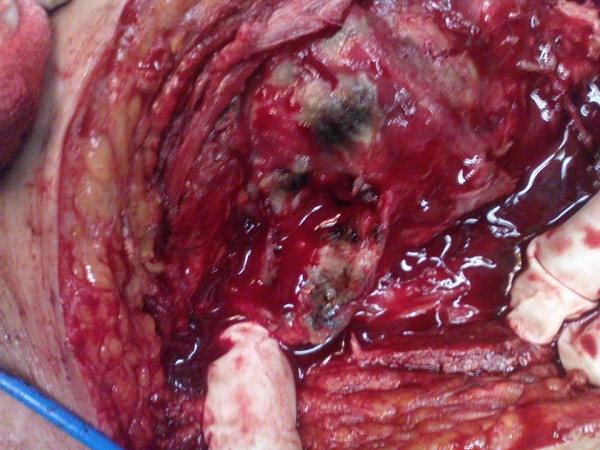
**Dark blue pigmentation of deep tissues and cartilage**. Photography of operation field and patient tissues, during operation.

Thus, before any definite treatment of fractured region, we decided to take biopsy from the lesion. After incision of skin and subcutaneous fat, there was a dark blue or even black lesion in the deep fascia and proximal portion of tensor fascia lata which had extended to deeper structures and joint capsule of hip joint (fig. 3).

We took a sample of the black tissue for pathologic exam and also another sample of the base of greater trochanteric region, which had a lytic appearance in x-ray of hip. The result of pathologic exam of these samples had shown nothing but ochronosis. In another operation, after about 2 weeks, we replaced the fractured hip with cemented hip prosthesis.

## Conclusion

**Figure 4:**
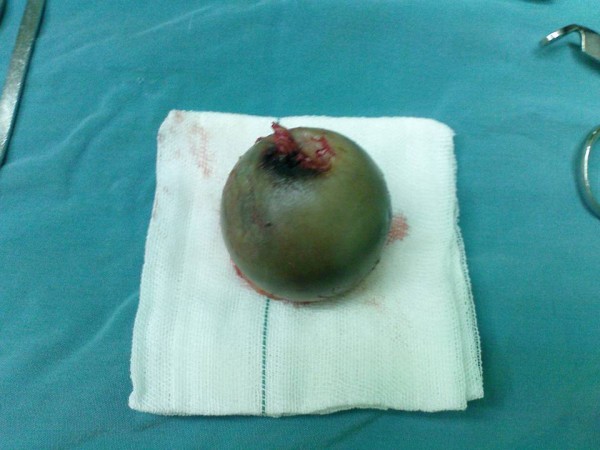
**Head of the femur**. Photography of operation field and patient tissues, during operation.

I had never imagined to view such a pigmentation until we prepared the patient for biopsy. There was a dark blue or even black material in the deep fascia of the thigh which extended toward deeper structures of hip joint. First, I thought it is an injection injury of oil or similar materials in deep structures of thigh and hip joint. But after discussing the issue with experts, and regarding their previous experiences, they suggested that it may be a metastasis of malignant melanoma. Since ochronosis is not a malignant disease, heroic resection (wide resection or radical resection) was not necessary. After approach to hip joint from posterior and after capsulotomy of hip, there was a dark blue pigmentation in the cartilage of femoral head (fig. 4) and acetabulum.

**Figure 5:**
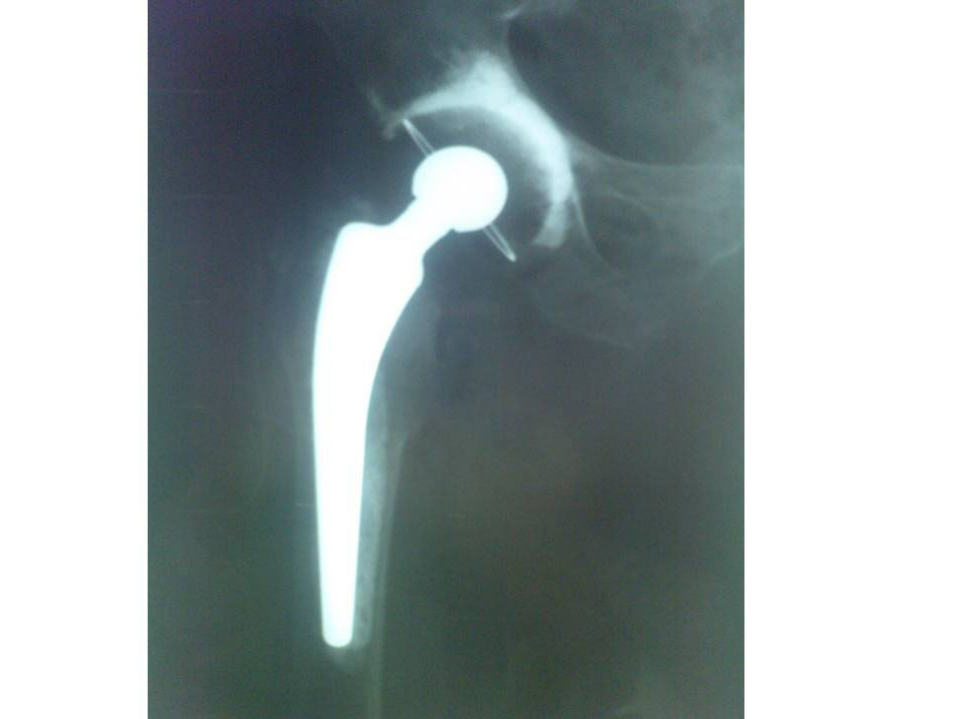
**Cemented total hip replacement prosthesis**. X ray, after operation.

Although she was only 54, we preferred not to preserve the head for osteosynthesis, because the cartilage of the femoral head was not healthy, the proximal part of femur had a lytic lesion, and the head fragment had porosis. Hence, there remained two other options: 1- bipolar prosthesis of hip joint; or, 2- total hip replacement. Because of the destructive nature of disease on connective tissues and cartilage of hip joint, the involvement of acetabular cartilage, and the probability of sooner destruction, we decided on a total hip replacement for her. In addition to lower price of cemented prosthesis, we decided not to use cementless prosthesis and pressfit constructs, because the bone quality was not good. At last, we came to this conclusion that a cemented total hip joint replacement is the final treatment for her (fig. 5).

## Consent

Written informed consent was obtained from the patient for publication of this case report and accompanying images. A copy of the written consent is available for review by the Editor-in-Chief of this journal.

## Competing interests

The authors declare that they have no competing interests.

## Authors' contributions

BS.: designer of study, responsible surgeon.

MJZ.: assistant surgeon.

EP.: Chief resident, assistant surgeon, main writer of the paper.

BS: corresponding author.

All authors read and approved the final manuscript.
